# The Impact of Post-Operative Phrenic Nerve Dysfunction on Lung Function Parameters and Long-Term Outcomes After Lung Transplantation

**DOI:** 10.3389/ti.2025.14691

**Published:** 2025-07-30

**Authors:** Keita Nakanishi, Caroline Hillebrand, Thomas Schweiger, Stefan Schwarz, Shahrokh Taghavi, Peter Jaksch, Alberto Benazzo, Toyofumi Fengshi Chen-Yoshikawa, Konrad Hoetzenecker

**Affiliations:** ^1^ Department of Thoracic Surgery, Medical University of Vienna, Vienna, Austria; ^2^ Department of Thoracic Surgery, Nagoya University Graduate School of Medicine, Nagoya, Japan; ^3^ Department of Thoracic Surgery, Vanderbilt University Medical Center, Nashville, TN, United States

**Keywords:** lung transplantation, phrenic nerve dysfunction, lung function parameters, diaphragmatic plication, surgery

## Abstract

A rare but important complication after lung transplantation (LTx) is postoperative phrenic nerve dysfunction (PND). Diaphragmatic plication (DP) is a well-established treatment option for PND, however, the long-term effect of PND and DP on lung function parameters and survival after LTx are currently unknown. We retrospectively reviewed 1400 LTx recipients transplanted at Medical University of Vienna between 01/2003 and 12/2022. Fluoroscopy and/or phrenic nerve conduction studies confirmed PND when chest radiographs after extubation showed a unilateral heightened diaphragm. We identified 25 patients with post-operative PND, of whom 12 underwent DP. The remaining 1,375 patients served as a control group. Median ICU-stay and hospital-stay were significantly longer in the PND groups (DP: 20 and 57 days; non-DP: 27 and 43 days; control group: 7 and 25 days; *P* = 0.001/*P* < 0.001). PND led to consistently lower %TLC in lung function tests performed within the first three years after LTx. DP was associated with lower %FEV1.0 early after LTx but it aligned to %FEV1.0 of the other groups during follow-up. Although PND significantly affected postoperative recovery after LTx, it did not impair long-term survival outcomes.

## Graphical Abstract

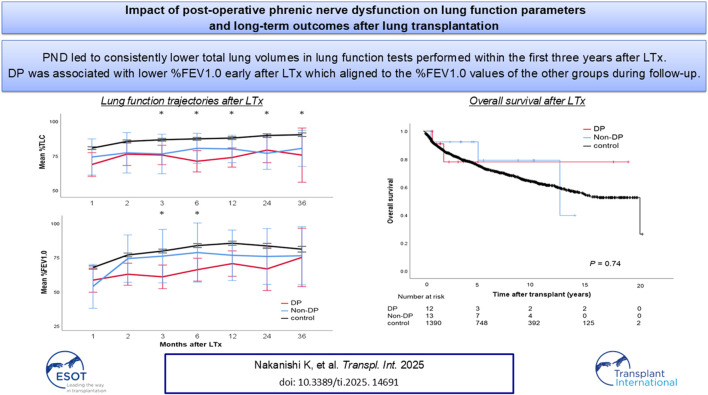

## Introduction

Lung transplantation (LTx) has evolved to a well-established treatment option for patients with end-stage pulmonary disease [1, 2]. A rare but important complication of LTx is postoperative phrenic nerve dysfunction (PND). The incidence of PND ranges from 3% to 9% after LTx, although it is more common in combined heart-lung transplant [3–5]. Mechanical injury of the phrenic nerve can be caused during pericardial manipulation, sternum retraction, and/or mediastinal dissection. Severe PND after LTx is associated with persistent lobar atelectasis, failure to clear airway secretions, and recurrent infections, potentially causing allograft dysfunction. PND has previously been shown to prolong post-operative intensive care unit- (ICU) and hospital-stay [6].

Diaphragmatic plication (DP) was first described in the 1980s as a surgical option for PND [7, 8]. The procedure is considered safe and effective in preventing atelectasis and improving symptoms in patients with PND. Recently, Lawrence et al. suggested two indications for DP in LTx recipients: DP for functional indications (symptomatic diaphragmatic dysfunction) and to overcome severe graft oversizing [9]. Other experiences of postoperative DP in patients receiving LTx are limited to case reports, mostly with favorable short-term results [10, 11]. However, the long-term effect of DP on lung function parameters and survival is still unknown.

Therefore, this study aimed to evaluate perioperative and long-term outcomes of LTx recipients with PND who received DP, LTx recipients with PND who did not receive DP, and a control group of LTx recipients with normal postoperative diaphragmatic function.

## Materials and Methods

### Study Population

**FIGURE 1:**
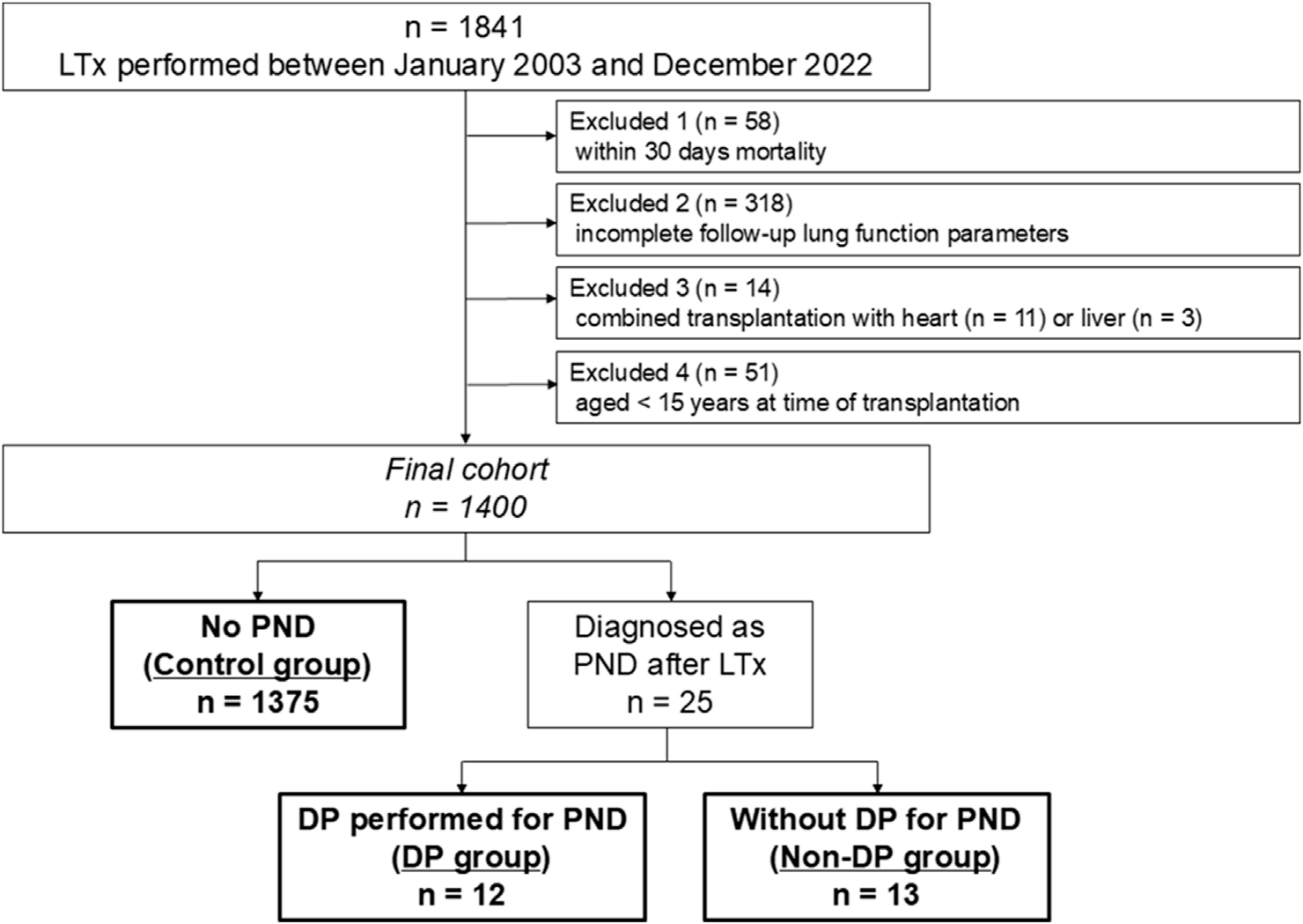
Patient selection. LTx, lung transplantation; PND, phrenic nerve dysfunction; DP, diaphragmatic plication.

Overall, 1,841 patients underwent LTx at the Medical University of Vienna between January 2003 and December 2022. As illustrated in Figure 1, the following patients were excluded: (i) patients who died within 30 days (n = 58), (ii) patients whose data according to lung function follow-up was incomplete due to transition to and follow-up in a local hospital close to the patients’ home (n = 318), (iii) patients who underwent combined transplantation (heart-lung, n = 11 or liver-lung, n = 3), and (iv) patients aged <15-year (n = 51). Size-reduction, single-lung transplantation, and lobar transplantation were not considered exclusion criteria. Eventually, 1,400 patients were included in the present study. Patients were divided into three groups: (i) patients who were diagnosed with PND after LTx and underwent DP (DP group), (ii) patients who were diagnosed with PND after LTx but did not undergo DP (non-DP group), and (iii) patients who did not develop PND after LTx (control group). PND was tested by fluoroscopy and/or phrenic nerve conduction studies (PNCS) whenever chest radiographs after extubation showed a unilateral heightened diaphragm. Phrenic nerve conduction studies were performed in accordance with the manufacturer’s instructions (Dantec Keypoint, Natus, Middleton, USA). Surface electrical stimulation was applied in the supraclavicular region, targeting the cervical portion of the phrenic nerve. Compound muscle action potentials were recorded at the costal margin along the anterior axillary line, typically between the 7th and 8th intercostal spaces, with the patient in a supine position and breathing spontaneously. As the procedure is non-invasive, we are not aware of any associated risks. Nerve conduction studies were conducted at the discretion of the treating physician. Regarding timing, nerve conduction testing is performed after weaning from the respirator, irrespective of the supplementary oxygen requirement. The indications for DP were (i) inability to wean from respirator or (ii) significant lower lobe atelectasis in computed tomography (CT) scans. The present study was approved by the ethics board on human research from the Medical University of Vienna (approval No. EK 1639/2023). Patient written consent for the publication of the study data was waived by the institutional ethics board due to the retrospective nature of the study.

### Data Collection

A review of medical charts, including preoperative examinations as well as intraoperative and postoperative data, from the hospital documentation system and the institutional transplant database was conducted. The outcome parameters analyzed were re-intubation and tracheostomy rates, ICU- and hospital-stay, % forced expiratory volume in one second (%FEV1.0) and % total lung capacity (%TLC) during follow-up lung function tests performed 1, 2, 3, 6, 12, 24 and 36 months after LTx as well as overall and Chronic lung allograft dysfunction (CLAD)-free survival. Pulmonary function tests were performed by certified pulmonary function technicians. %FEV1.0 and %TLC were calculated as the ratio of actual to predicted values using European Respiratory Society formulas [12], which are as follows:

FEV1.0:

Male: 4.3*height (m) - 0.029*age - 2.49.

Female: 3.95*height (m) - 0.025*age - 2.6.

TLC:

Male: 7.99*height (m) - 7.08.

Female: 6.60*height (m) - 5.79.

### Surgical Procedure

Organ procurements and transplant procedures were performed according to the standardized institutional protocol published elsewhere [13, 14]. Donor lungs were perfused with low-potassium dextran solution and stored on ice, as moderate hypothermic storage at 10° was only established after 2022 in our institution. *Ex vivo* lung perfusion (EVLP) was performed in selected cases. For bilateral lung transplantation, a clamshell incision or bilateral anterior thoracotomies in the fourth intercostal space were made. Single-lung transplantation was performed through an anterolateral thoracotomy. Slightly oversized grafts were tailored by extra anatomical downsizing of the middle lobar and/or lingular resection. The need for downsizing was ultimately decided by the implantation team before closing the chest. Basic immunosuppression consisted of a triple-drug regimen with cyclosporine (or tacrolimus), mycophenolate mofetil, and corticosteroids. Alemtuzumab or anti-thymocyte globulin was used for induction therapy in most recipients. DP was performed as previously published based on the common principle of lowering the entire diaphragmatic dome by suturing the redundant part from the posterior costophrenic angle to the cardio-phrenic angle [15] at a median of 27 days (IQR, 15–125) after LTx. The procedure was carried out via a separate lateral or posterolateral thoracotomy through the 6th intercostal space, distinct from the original transplant incision.

### Statistical Analysis

All statistical analyses of data were performed using the SPSS Statistics 25 software (IBM Corporation, Armonk, NY). Categorical variables were compared using the Fisher’s exact test. For continuous variables, the Student’s t-test was used. One-way analysis of variance (ANOVA) and the Kruskal-Wallis test were applied to compare the means or medians of more than two samples, respectively. For the comparison of pulmonary function parameters among the groups, one-way ANOVA was performed. Overall survival (OS) and CLAD-free survival were analyzed by the Kaplan-Meier method, and log-rank tests were used to compare survival. OS was defined as the time from surgery to death due to any cause. CLAD-free survival was defined as the time from surgery to either the first development of CLAD or death due to any cause. For all analyses, a *P*-value of <0.05 was considered statistically significant.

## Results

We identified 25 patients with PND (1.8%), of whom 12 underwent DP. The remaining 1,375 patients served as a control group. Donor characteristics are summarized in Table 1. There were no significant differences in terms of age, sex, height, blood type, cause of death, smoking history, and total intubation days observed between the three groups. Last PaO2 at 1.0 FiO2 was slightly worse in the DP group compared to the non-DP and control group (median, 400.6 mmHg [IQR, 351.2–480.5] vs. 438.0 mmHg [IQR, 360.0–520.1] vs. 439.0 mmHg [IQR, 373.5–506.0]), but the difference did not reach significant (*P* = 0.54).

**TABLE 1:** Donor characteristic.

		DP group (n = 12)	Non-DP group (n = 13)	Control group (n = 1,375)	*P* value
Age, years	Median (IQR)	49 (33–60)	41 (31–51)	44 (31–53)	0.433
Sex female: male ratio	n (%)	7:5 (58:42)	7:6 (54:46)	681:694 (50:50)	0.781
Height, cm	Median (IQR)	170 (168–180)	170 (165–178)	170 (165–180)	0.554
Blood group	n (%)				0.480
O		2 (17)	5 (38)	552 (40)	
A		8 (66)	5 (38)	578 (42)	
B		2 (17)	3 (24)	179 (13)	
AB		0	0	66 (5)	
Donation type	n (%)				0.466
DBD		11 (92)	13 (100)	1,317 (96)	
DCD		1 (8)	0	58 (4)	
Cause of death	n (%)				0.367
Cerebrovascular/Stroke		8 (66)	8 (61)	851 (62)	
Anoxia/Cardiac arrest		2 (17)	0	42 (3)	
Trauma		2 (17)	3 (23)	345 (25)	
Suicide		0	1 (8)	41 (3)	
Others		0	1 (8)	96 (7)	
Smoking history	n (%)				0.156
Yes		5 (42)	6 (46)	649 (47)	
No		6 (50)	7 (54)	429 (31)	
Unknown		1 (8)	0	297 (22)	
Total intubation days	Median (IQR)	3 (3–11)	3 (2–5)	3 (1–5)	0.101
Last PaO2 at 1.0 FiO2, mmHg	Median (IQR)	400.6 (351.2–480.5)	438.0 (362.0–520.1)	439.0 (373.5–506.0)	0.539
Last PaCO2 at 1.0 FiO2, mmHg	Median (IQR)	39.4 (35.9–43.0)	39.8 (34.9–41.4)	39.0 (35.0–42.9)	0.933

DP, diaphragmatic plication; IQR, interquartile range; DBD, donor after brain death; DCD, donor after circulatory death.

Basic recipient demographic data and surgical characteristics of the three study groups are provided in Tables 2, 3. There were no significant differences in sex, height, diagnosis, extracorporeal life support bridge-to-LTx, lung allocation score, and type of LTx. Patients of the non-DP group were significantly younger compared to patients of the DP and control group (median, 48 years [IQR, 31–60] vs. 59 years [IQR, 56–66] vs. 54 years [IQR, 39–60]; *P* = 0.039). Chronic obstructive pulmonary disease was the most common indication for LTx in all three groups (DP: 59%, non-DP: 39%, control: 40%). DP was performed more often in later years (*P* = 0.04). Single-lung transplantations were in general rarely performed and only found in the control group (8%). Two cases of lung retransplantation were included in the control group. Size reduction of the donor lungs was performed most frequently in the DP group compared to others (58% vs. 38% vs. 35%) (*P* = 0.014). Most patients underwent transplantation with the use of central venoarterial extracorporeal membrane oxygenation (VA-ECMO), with 92% in both the DP and non-DP groups, and 77% in the control group (Table 3). There were no significant differences in terms of total preservation time, duration of surgery, intraoperative transfusions, and induction therapy between the three groups.

**TABLE 2:** Recipient characteristics.

		DP group (n = 12)	Non-DP group (n = 13)	Control group (n = 1,375)	*P* value
Age, years	Median (IQR)	59 (56–66)	48 (31–60)	54 (39–60)	0.039
Female: male ratio	n (%)	3:9 (25:75)	6:7 (44:56)	602:773 (44:56)	0.488
Height, cm	Median (IQR)	175 (165–178)	168 (159–179)	170 (163–176)	0.409
Diagnosis	n (%)				0.674
COPD		7 (59)	5 (39)	548 (40)	
Fibrosis		3 (25)	2 (15)	325 (24)	
Cystic fibrosis		0	3 (23)	228 (16)	
PAH		1 (8)	1 (8)	70 (5)	
Others		1 (8)	2 (15)	204 (15)	
ECLS bridge-to-LTx	n (%)	2 (17)	1 (8)	98 (7)	0.261
LAS	Median (IQR)	37.1 (32.7–61.3)	38.6 (32.7–42.4)	35.4 (32.3–43.4)	0.728
Transplant era	n (%)				0.040
2003–2009		2 (17)	2 (15)	399 (29)	
2010–2016		1 (8)	5 (39)	514 (37)	
2017–2022		9 (75)	6 (46)	462 (34)	
Type of LTx	n (%)				0.702
Double-lung		12 (100)	13 (100)	1,270 (92)	
Single-lung		0	0	105 (8)	

DP, diaphragmatic plication; IQR, interquartile range; COPD, chronic obstructive pulmonary disease; PAH, pulmonary arterial hypertension; ECLS, extracorporeal life support; LTx, lung transplantation; LAS, lung allocation score.

**TABLE 3:** Transplant procedure.

		DP group (n = 12)	Non-DP group (n = 13)	Control group (n = 1,375)	*P* value
EVLP	n (%)	0	2 (15)	58 (4)	0.186
Approach	n (%)				0.109
Clamshell		7 (58)	12 (92)	935 (68)	
Thoracotomy		5 (42)	1 (8)	440 (32)	
Size reduction	n (%)				0.014
Whole lungs		4 (34)	7 (54)	765 (55)	
Extra-anatomical size reduction		7 (58)	5 (38)	477 (35)	
Lobar		0	1 (8)	133 (10)	
Others		1 (8)	0	0	
Type of intraoperative support	n (%)				0.552
No support		1 (8)	1 (8)	298 (22)	
Intraoperative ECMO		11 (92)	12 (92)	1,056 (77)	
CPB		0	0	21 (1)	
Total preservation time 2nd lung, min	Median (IQR)	410 (364–508)	455 (381–513)	386 (338–450)	0.071
Duration of surgery, min	Median (IQR)	338 (292–405)	300 (248–436)	295 (250–349)	0.100
Intraoperative transfusions					
RBC units	Median (IQR)	3 (2–5)	6 (3–8)	4 (2–6)	0.283
FFP concentrates	Median (IQR)	8 (5–15)	10 (6–12)	9 (5–12)	0.625
Induction	n (%)				0.250
Yes		6 (50)	8 (62)	960 (70)	
No		6 (50)	5 (38)	415 (30)	

DP, diaphragmatic plication; EVLP, *ex vivo* lung perfusion; ECMO, extracorporeal membrane oxygenation; CPB, cardiac pulmonary bypass; IQR, interquartile range; RBC, red blood cell; FFP, frozen fresh plasma.

**FIGURE 2:**
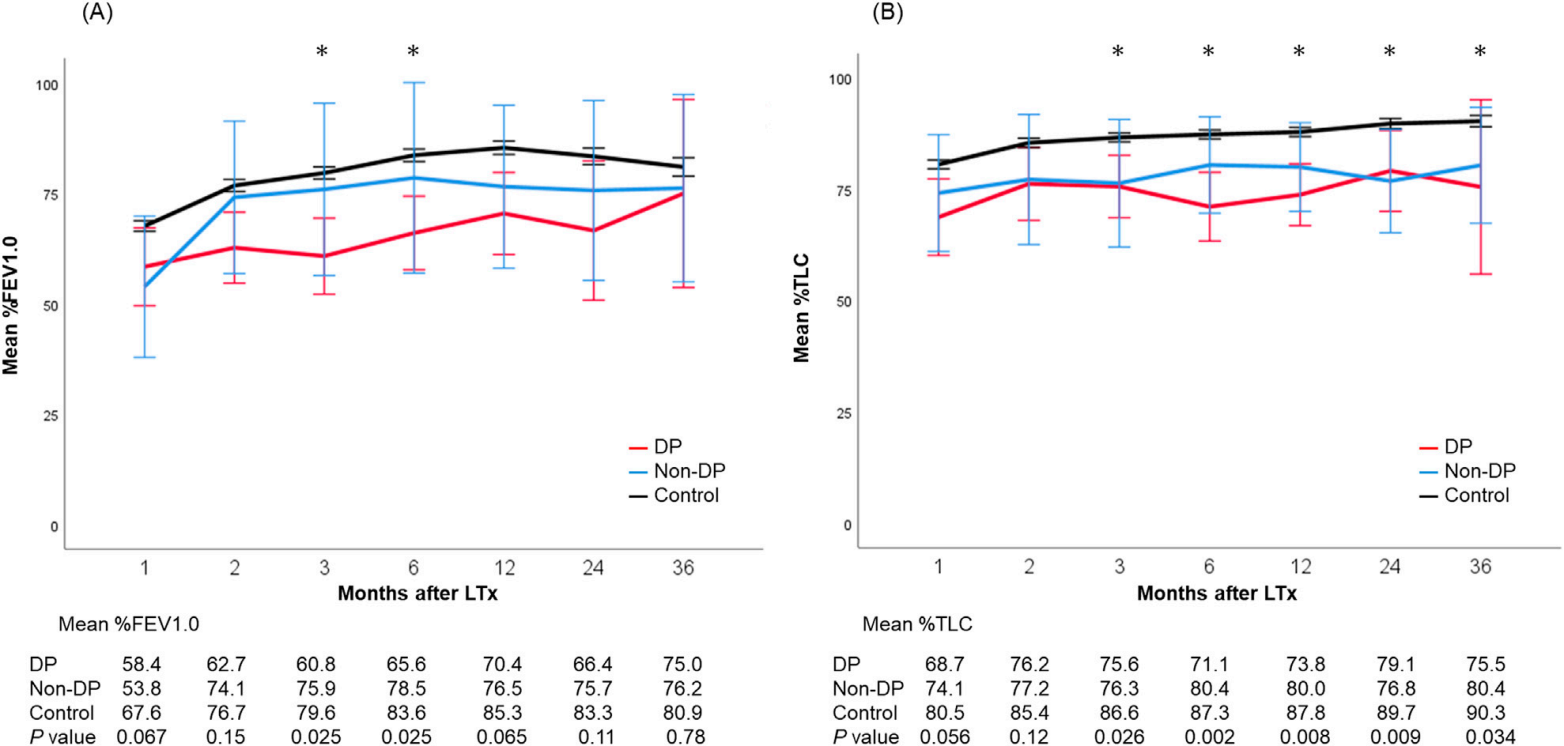
Trajectories of lung function parameters including mean %FEV1.0 **(A)** and %TLC **(B)** after lung transplantation. Each bar shows the standard error of the mean. *, <0.05. DP, diaphragmatic plication.

**FIGURE 3:**
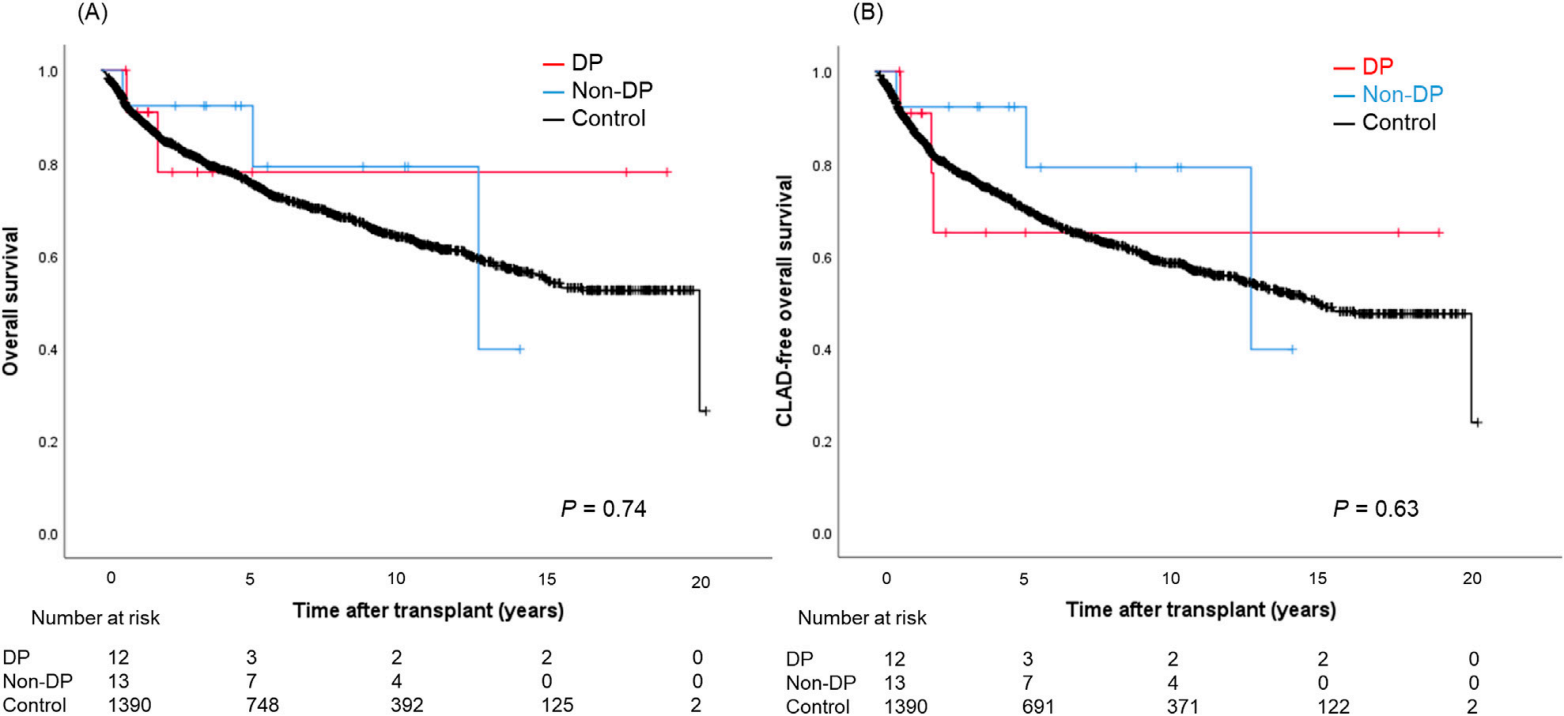
Overall survival **(A)** and CLAD-free survival **(B)** curves in patients with the DP group, the non-DP group, and the control group. DP, diaphragmatic plication; CLAD, chronic lung allograft dysfunction.

Postoperative outcomes are presented in Table 4. There was no difference in laterality of PND between DP and non-DP patients (*P* = 0.75). Both, median ICU-stay as well as hospital-stay, were significantly longer in the PND groups (DP: 20 [IQR, 9–57] and 57 [IQR, 23–93] days; non-DP: 27 [IQR, 6–38] and 43 [IQR, 29–60] days; control group: 7 [IQR, 4–15] and 25 [IQR, 19–36] days; *P* = 0.001/*P* < 0.001). Re-intubation rate was the highest in the non-DP group compared to the DP and control group (62% vs. 33% vs. 17%, *P* < 0.001) as well as the tracheostomy rate (54% vs. 33% vs. 20%, *P* = 0.005). Trajectories of lung function parameters are shown in Figure 2. % FEV1.0 at three and six months after LTx was significantly worse in the DP group compared to the other groups. However, it gradually improved over time, and there was no significant difference any longer between the three groups 36 months after LTx. In contrast to this, the measured %TLC remained consistently lower in the PND groups. Of note, the %TLC was not different between patients of the DP and non-DP groups. The 5-year OS/CLAD-free survival rates were 77.9%/64.9% in the DP group, 92.3%/92.3% in the non-DP group, and 75.7%/70.3% in the control group (*P* = 0.74/*P* = 0.63) (Figures 3A,B).

**TABLE 4:** Outcome parameters.

		DP group (n = 12)	Non-DP group (n = 13)	Control group (n = 1,375)	*P* value
Localization of PND	n (%)				0.751
Right		6 (50)	6 (46)	-	
Left		4 (33)	6 (46)	-	
Bilateral		2 (17)	1 (8)	-	
Time from LTx to plication, days	Median (IQR)	27 (15–125)	-	-	
Tracheostomy	n (%)	4 (33)	7 (54)	268 (20)	0.005
Re-intubation	n (%)	4 (33)	8 (62)	228 (17)	<0.001
ICU-stay, days	Median (IQR)	20 (9–57)	27 (6–38)	7 (4–15)	0.001
Hospital-stay, days	Median (IQR)	57 (23–93)	43 (29–60)	25 (19–36)	<0.001
5-year overall survival	%	77.9	92.3	75.7	0.742
5-year CLAD-free survival	%	64.9	92.3	70.3	0.633

DP, diaphragmatic plication; ICU, intensive care unit; PND, phrenic nerve dysfunction; LTx, lung transplantation; IQR, interquartile range; CLAD, chronic lung allograft dysfunction.

## Discussion

This study demonstrated that PND–despite having a significant impact on perioperative recovery–did not impair long-term overall and CLAD-free survival after LTx. This is well in line with trajectories of lung function tests, showing lower %FEV1.0 and %TLC early after LTx. Interestingly, %FEV1.0 seems to recover over time, whereas %TLC remained lower in non-DP and DP patients compared to the control group. To the best of our knowledge, this is the first study to investigate lung function parameters and long-term outcomes of LTx recipients with PND and DP.

The true incidence of PND after LTx is not clear. This complication was first systematically examined in LTx recipients in 1995, with a reported incidence as high as 29% [16]. Subsequent series have reported much lower rates ranging from 3% to 9% [3, 5]. PND was observed in only 1.8% of our patients. This variability in post-transplant PND might be explained by the heterogeneity of underlying diseases, improvements in surgical technique, as well as differences in the definition and diagnostic methods used. The most recent study of PND after LTx was published by the Santander Lung Transplant Program. In a well-conducted prospective observational study covering over 4 years, the group could show that some degree of diaphragmatic impairment determined by systematic phrenic nerve conduction studies was evident in 43.3% of subjects and 29.0% of operated hemithoraces [17]. The main risk factors identified by this study were female gender, double-LTx, right grafts, clamshell incisions, and mediastinal adhesions. Morbidity was increased in PND without any difference in mortality. The significant lower numbers of PND in our cohort might be attributed to the fact that only patients with an elevated diaphragm were tested, thus, mild or temporary dysfunctions were not captured.

Several surgical principles have to be respected to avoid injury to the phrenic nerve. When the chest is opened by a clamshell incision, the anterior mediastinum should be mobilized to reduce the tension on the nerves. Pushing the heart should be reduced to a minimum, as the nerve can also be damaged by extensive pressure. Any dissection close to the nerve should be done with scissors or in a blunt way. The use of cautery should be limited to a minimum. Intraoperative electrophysiological phrenic nerve monitoring, which has previously been tested during cardiac surgery, could also be a tool to prevent damage to the phrenic nerve but its applicability in LTx needs to be determined [18].

The limited number of published series on PND after LTx found an increased length of ICU-stay, increased readmission rates to the ICU, and increased duration of hospitalization [3, 4, 6, 17]. Studies on the long-term effects of PND on lung function parameters are scarce. Lawrence et al. reported that patients with DP had consistently lower FEV1.0 than those without DP at 1-, 2-, and 3-year post-LTx although the gap between the two groups appeared to remain stable [9]. Furthermore, 1-, 2-, and 3-year survival as well as 3-year CLAD-free survival were similar. In our cohort, %FEV1.0 was significant lower early after LTx in DP patients compared to the other groups. Interestingly, %FEV1.0 seemed to improve over time. %TLC was significantly worse in both PND groups compared to the values of the control group. Furthermore, there was no difference in terms of %TLC between DP and non-DP patients. An important caveat of this interpretation is that patients in the non-DP group were significantly younger and patients in the DP group were transplanted in the most recent era (2017–2022). These bias may account for differences in the long-term lung function trajectories.

DP is a well-established treatment option for PND and is frequently performed in non-LTx patients. Freeman et al. conducted a single-center retrospective study to assess the impact of DP on the functional and physiologic outcomes in symptomatic patients [19]. In this study, mean FEV1.0 and TLC improved by 23% and 19%, respectively, when measured 6 months after surgery. The authors concluded that DP significantly improved pulmonary function, symptoms of dyspnea, and patient functional status. We performed DP liberally in patients with a confirmed PND and difficulties weaning from respirator or significant lower lobe atelectasis in CT scans. The optimal timing for DP after LTx remains unclear due to limited evidence in the literature. In our study, DP was performed at a median of 27 days post-transplant (IQR: 15–125 days). To our knowledge, only one previous study has reported the timing of DP, with a median of 16 days (range: 1–34 days), but it did not evaluate long-term outcomes [9]. Our study is the first to investigate the long-term impact of DP on pulmonary function after LTx. Based on our limited experience, we believe that DP should be considered when patients demonstrate persistent diaphragmatic dysfunction that interferes with respirator weaning or leads to significant atelectasis. If the patient’s general condition, including wound healing and immunosuppression status, is acceptable, proceeding with DP within the first month appears to be reasonable. Furthermore, early DP may contribute to improved respiratory outcomes, as it facilitates weaning in patients previously unweanable from mechanical ventilation.

An interesting finding from long-term lung function trajectories is that FEV1 slightly improved in our DP patients. It is known that the function of accessory muscles of respiration improved by the rehabilitation after LTx. These muscles can compensate for the loss of diaphragmatic function and prevent detrimental long-term sequelae. Therefore, post-LTx rehabilitation is an essential part of successful lung transplant programs. Pulmonary rehabilitative exercises focus on restoring the strength and function of the diaphragm [20]. Furthermore, inspiratory muscle training can be performed to improve diaphragmatic weakness due to PND and is an important treatment option, particularly in cases of prolonged mechanical ventilation [21].

This study has several limitations. First, this retrospective study introduces several potential biases. There might have been a selection bias, particularly in pulmonary function tests, in which patients who have a longer survival have better function results. Second, the numbers of patients in the DP and non-DP groups are low, which may have limited the power to detect statistically significant differences. Due to the small sample size, we were also unable to perform multivariate analyses to adjust for potential confounding factors, including recipient age, transplant year, and diagnosis. Moreover, we might have missed some cases due to the exclusion criteria of 30-day mortality. However, this study aimed to examine long-term outcomes and lung function trajectories. Third, this study cannot account for surgical improvements, advances in perioperative care, and innovations in immunosuppressive therapy during the long study period of almost 20 years. Furthermore, we did not collect detailed information on patients’ history of prior thoracic surgery or the presence of preoperative PND. Previous thoracic surgeries, such as lung resections or cardiac operations, could potentially cause phrenic nerve injury, and it is possible that some patients had impaired phrenic nerve function before LTx. Although we excluded cases of combined heart-lung transplantation based on the prior report [4] indicating a higher incidence of phrenic nerve injury in such procedures, we did not systematically screen for other types of prior thoracic surgical interventions. Therefore, we cannot entirely exclude the possibility that some of the postoperative phrenic nerve dysfunction observed in this study may have originated from preexisting conditions. Subsequent studies from other high-volume LTx centers or even a multicenter approach are warranted to confirm and validate our findings in independent patient cohorts.

In conclusion, we demonstrated that PND was associated with complicated recovery after LTx. PND led to slightly but consistently lower total lung volumes in lung function tests performed within the first three years after LTx. Despite this, PND was not associated with impaired long-term and CLAD-free survival.

## Data Availability

The raw data supporting the conclusions of this article will be made available by the authors, without undue reservation.
